# Supportive Oligonucleotide Therapy (SOT) as a Potential Treatment for Viral Infections and Lyme Disease: Preliminary Results

**DOI:** 10.3390/idr14060084

**Published:** 2022-11-03

**Authors:** Panagiotis Apostolou, Aggelos Iliopoulos, Georgios Beis, Ioannis Papasotiriou

**Affiliations:** 1 Research Genetic Cancer Centre S.A., Industrial Area of Florina, 53100 Florina, Greece; 2 Research & Development, Research Genetic Cancer Centre International GmbH, 6300 Zug, Switzerland

**Keywords:** antisense therapy, Lyme disease, Epstein–Barr virus, herpes simplex viruses, inferential statistics

## Abstract

Antisense therapy is widely used as an alternative therapeutic option for various diseases. RNA interference might be effective in infections, through the degradation of messenger RNA and, therefore, translation process. Hence, proteins essential for microorganisms and viruses’ proliferation and metabolism are inhibited, leading to their elimination. The present study aimed to evaluate the use of oligonucleotide in patients infected by Epstein–Barr (EBV) or Herpes Simplex Viruses 1/2 or with Lyme Disease caused by Borrelia burgdorferi. Blood samples were collected from 115 patients and the different species were characterized using molecular biology techniques. Then, SOT molecules (Supportive Oligonucleotide Therapy), which are specific small interfering RNA (siRNA), were designed, produced, and evaluated, for each specific strain. Oligonucleotides were administered intravenously to patients and then a quantitative Polymerase Chain Reaction was used to evaluate the effectiveness of SOT. This study revealed that for Lyme Disease, one or two SOT administrations can lead to a statistically significant decrease in DNA copies, while for viruses, two or three administrations are required to achieve a statistically significant reduction in the genetic material. These preliminary results indicate that antisense SOT therapy can be considered a potential treatment for viral as well as Lyme diseases.

## 1. Introduction

Antisense therapy, using nucleic-acid-based oligonucleotides, is widely used in the treatment of various diseases, including cancer, viral, or general infections, as well as genetic disorders. The mechanism of action is usually through RNA-mediated interference (RNAi), where short doubled-stranded RNA (dsRNA) molecules cause messenger RNA (mRNA) degradation [1]. RNA interference is a physiological process, required for the regulation of gene expression. Once a peptide (protein) is required for a cell, then the process of transcription takes place and mRNA molecules for a specific gene are produced. These molecules can then be translated to peptides (proteins), which, in turn, proceed to the desired function. Upon the completion of this function, the production needs to be stopped; therefore, the existing mRNA molecules are not translated. Through the RNAi mechanism, the existing mRNA molecules for this gene are degraded; therefore, the peptide (protein) production is interrupted. The RNAi process begins with dsRNA molecules, produced either by cells or inserted into them. Then, the dsRNA molecules, either endogenic or extragenic, are fragmented into small interfering RNA (siRNA) molecules, which, in turn, denatured to single-stranded RNA, complementary to a unique sequence of mRNA. The single-stranded siRNA then binds to the mRNA, in a specific position and, finally, the RNA-induced silencing complex (RISC) leads to the degradation of mRNA. Therefore, mRNA cannot be correctly translated, leading either to inappropriate peptide or no peptide production [2].

Preliminary results of a recent study showed that supportive oligonucleotide therapy (SOT) can be beneficial for cancer patients, used either as monotherapy or in combination with other therapies, irrespective of the cancer type. The number of Circulating Tumor Cells (CTCs) in patients was reduced post-SOT administration, while patients’ clinical status improved as well [3]. In addition, RNAi-based therapy has been used for many years against viruses, such as human immunodeficiency virus (HIV), hepatitis B virus (HBV), or respiratory viruses.

Silencing DC-specific intercellular adhesion molecule 3-grabbing nonintegrin, which is involved in signaling, led to the inhibition of the attachment of gp120 envelope protein of HIV to them and, therefore, affected the transfer of HIV to T cells [4]. In HBV, siRNA molecules contributed to the inhibition of genes, important for replication [5]. Studies in respiratory syncytial virus (RSV), parainfluenza virus (PIV), and influenza virus (an Orthomyxovirus) demonstrated that intranasal siRNAs might be beneficial against the above viruses [6]. Furthermore, antisense therapy is also applicable to bacterial infections, particularly in cases of antibiotic-resistant strains. siRNA molecules might also be used to inhibit the genes involved in resistance, contributing to further treatment with conventional antibiotics [7]. In addition, RNA interference has been proven to be potentially beneficial for tick-borne diseases, since dsRNA molecules can be introduced to ticks and lead to a reduction in specific gene expression [8].

In the present study, we aimed to evaluate the efficacy of support oligonucleotide therapy in patients infected by Epstein–Barr (EBV) or Herpes Simplex Viruses 1/2, as well as individuals with Lyme Disease. Targeting of the zta gene or other lytic proteins of EBV contributed to the inhibition of EBV replication, while inhibition of latent membrane protein (LMP-1) led to apoptosis of EBV-immortalized cells [9,10]. Apart from LMP-1, the nuclear antigen 1 (EBNA-1) could also be targeted, as it has also demonstrated that the proliferation of EBV-immortalized B cells is inhibited upon EBNA-1 antisense oligonucleotides [11]. RNA interference technology is also applicable to ticks in the last few decades, while recent research demonstrated that small RNAs, produced by RNA-dependent RNA Polymerases (RdRPs), regulate gene expression [12,13].

SOTs were individually designed, based on each strain detected in every patient, and specific gene, important for their amplification of metabolism and administered intravenous (IV). SOTs are used as a single dose, or repeated, depending on the follow-up data. Genetic material was measured with quantitative polymerase chain reaction (qPCR), while the clinical status of every patient was also evaluated post-administration of the modified oligonucleotides. 

## 2. Materials and Methods

### 2.1. Samples

The study included 115 patients of both genders infected either with EBV (59 patients), HSV1 or HSV2 (28 patients), or Lyme Disease (28 patients). The sample distribution was random including samples from Europe and the USA. The patients included exhibited positive immunology results (IgG-IgM antibodies), which were then confirmed with qPCR in our facilities. All patients received the appropriate oligonucleotide therapy, based on their infection, while patients who did not receive SOT were not included. All methods were carried out following relevant guidelines and regulations.

The study was reviewed and approved by the Bioethical Committee of the Research Genetic Cancer Centre Group. All patients provided written consent for the use of their samples in the present study. The patients retained the right to withdraw their samples until the date when the sample was received at the laboratory and tested.

### 2.2. Strain Detection

DNA was isolated from both cells and serum of blood samples, with QIAamp DNA Mini Kit (51306; Qiagen, Hilden, Germany) and qPCR performed for HSV1, HSV2, EBV, and Borrelia burgdorferi (Lyme Disease), with specific primers. Primers included genes of interest for all the different microorganisms and viruses, as well as universal primers for general infection (e.g., 16SrRNA for Borrelia). For EBV, primers included the detection of viral-capsid antigen (VCA), nuclear antigen (EBNA), as well as early antigen (EA). Primers of HSV1 and HSV2 focused on glycoprotein G1 (gG1) and glycoprotein G2 (gG2). Finally, the detection of Lyme Disease is based on primers targeting outer-surface proteins (for example, OspA-OspB). All the primers were designed on Beacon Designer 8 and obtained by WelbioAps (Zug, Switzerland). Primer sequences were evaluated using the Basic Local Alignment Search Tool (https://blast.ncbi.nlm.nih.gov/Blast.cgi, accessed on 17 March 2022) searches to exclude primers that would amplify undesired strains. Primer sequences are presented in Table 1.

Samples were analyzed by PCR as follows: 95 °C for 2 min, followed by 40 cycles of 95 °C for 10 s and 59 °C for 30 s. Upon amplification, a melting curve analysis was performed from 70 °C to 90 °C at 0.5 °C increments for 5 s at each step. In all reactions, the same amount of DNA was used. In addition, appropriate positive and negative controls were used and all reactions were performed in triplicates. Positive controls included DNA from EBV (VR-3247SD; LGC Standards GmbH, Wesel, Germany), HSV1 (VR539DQ; LGC Standards GmbH), HSV2 (VR-540DQ; LGC Standards GmbH), and Borrelia burgdorferi (35210D-5; LGC Standards GmbH). qPCR data were exported as “Threshold Cycle” or “Ct”. Ct > 35 indicated negative, while Ct < 35 was positive. The higher the Ct value, the lower the DNA copies for the specific microorganism.

### 2.3. SOT Production—Administration

Following strain detection, modified double-stranded RNA molecules, complementary to a specific gene, were designed and produced in the Oligomaker48 DNA/RNA Synthesizer. EBV and HSV1/2 molecules targeted genes important for viruses’ replication, while for B. burgdorferi, the siRNA targeted the lipoprotein gene. Then, dsRNA molecules were purified with High-Performance Liquid Chromatography (HPLC), tested for pathogens and endotoxins, and finally freeze-dried. Before administration, SOT was reconstituted in 1 ml of water for injection and dissolved on a syringe and added 9 ml of water for injection. The final solution was applied intravenously.

### 2.4. SOT Questionnaire

The questionnaire used in the present study involved information concerning patients’ date of birth and gender, as well as the date of the last dose of the respective treatment followed. Each dose was administered within the time interval of 4–6 months after the previous one.

### 2.5. Statistical Methods

This study involves a design of repeated measurements (design within subjects), in which study participants are measured for n > 2 times in the same dependent variable. Particularly, repeated measurements occur when a study participant is exposed to two or more experimental or therapeutic conditions (i.e., factor levels), leading to correlations between outcome measurements [18]. 

The traditional procedure for testing hypotheses in such a dataset is the one-way repeated-measures ANOVA [19], which is an extension of the paired T-test and compares mean values for three or more groups [20]. This model is used to evaluate differences in an outcome between the same subjects but at different time intervals. In the ANOVA test, the null hypothesis presupposes that all relative means are equal. However, it cannot determine which group means are equal or not. Therefore, relevant post hoc tests must be performed and, more specifically, multiple null hypotheses testing, in which the significance criterion is adjusted accordingly. In addition, the hypothesis of sphericity has to be tested during the calculation of the ANOVA test. Particularly, the Mauchly test can be used to evaluate the sphericity hypothesis and the Geisser–Greenhouse sphericity correction can be applied to factors that violate the sphericity hypothesis [21,22]. Specifically, when the sphericity hypothesis is violated, adjustments have to be made to the respective degrees of freedom, affecting the statistical significance (i.e., *p*-value) of the test. In this study, the correction is applied by multiplying the degrees of freedom (DFn and DFd) with the correction Greenhouse–Geisser (GG) epsilon values. Epsilon (ε) provides a measure of the degree to which sphericity has been violated. The value 1 indicates no deviation from the sphericity (all variations in the group differences are equal), while a violation of sphericity results in an epsilon value below 1. 

However, the ANOVA test is based on the assumption that the populations throughout the sample are distributed normally. The Shapiro–Wilk normality test [23] was utilized to check the normality assumption in this study. In case the dataset does not satisfy normality, the Friedman test [24,25] was applied, as a non-parametric version of ANOVA for dependent samples. In such a case, the Wilcoxon signed-rank test is used as a post hoc test [26]. In this study, for the adjustment of p-values, the Bonferroni method [27] was used for both parametric as well as non-parametric tests.

**Figure 1:**
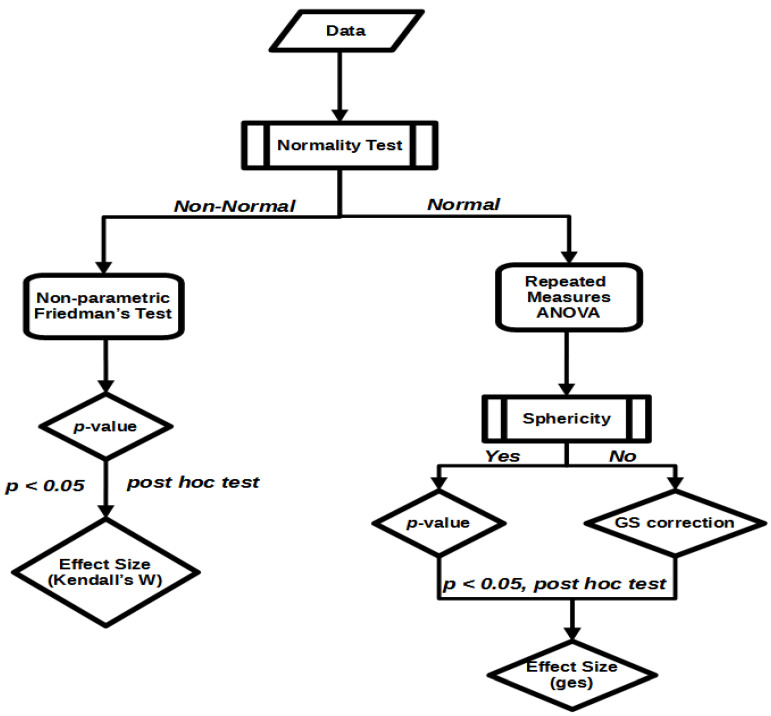
The flowchart of the statistical methodology and respective procedures considered in this study.

Finally, effect sizes were also estimated for checking if SOT therapies have an effect greater than zero and/or how large these effects are. Reporting effect sizes is useful for three reasons, because they [28]: (a) represent the magnitude of the reported effects in a standardized way, allowing for a practical significance of obtained results, (b) can be used for meta-analytic conclusions by comparing results across studies, and (c) constitute the base for planning a new study. In this study, the generalized eta squared (ges) was calculated as a measure of the magnitude of the ANOVA test, while for the Friedman test, Kendall’s W effect size was evaluated [29]. An overview of the statistical methodology applied in this study is provided in Figure 1. 

The statistical analysis of this study was performed using the statistical software R [30] and R packages, such as tidyverse package [31] and ggpubr package [32], for data handling and visualization, as well as rstatix package [33] for statistical analysis.

## 3. Results

### 3.1. Lyme Disease

For Lyme disease, Ct measurements (Cts) were obtained for three consecutive time points, concerning 28 individuals with a median age of 51 years. The application of the Shapiro–Wilk test revealed that the criterion of normality is not met (*p*-value= 5.84 × 10^−6^ < 0.05). Therefore, since the ANOVA test cannot be used, the Friedman rank test, a non-parametric version of ANOVA, as well as the Wilcoxon signed-rank test (for multiple pairwise comparisons) were utilized. The results, depicted in Table 2, revealed statistically significant differences at different time points, with *p* = 1.28 × 10^−8^ (<0.05) and test statistic X2(2) = 36.35. For the investigation of what pairs of Cts’ means were significantly different, a multiple comparison test was conducted and the results are represented in Table 2, which shows the adjusted p-values (Bonferroni adjustment). In particular, the pairwise Wilcoxon signed-rank test revealed statistically significant differences in Cts, between t0 and t1 pair’s comparison (*p* = 3.12 × 10^−5^ < 0.05), t0 and t2 pair’s comparison (*p* = 6.87 × 10^−6^ < 0.05) and t1 and t2 pair’s comparison (*p* = 2.00 × 10^−3^ < 0.05). Finally, Kendall’s W was used as a measure of the effect size of Friedman’s test. In this case, a large effect size was detected, namely, Kendall W = 0.65 (Table 2).

**Figure 2:**
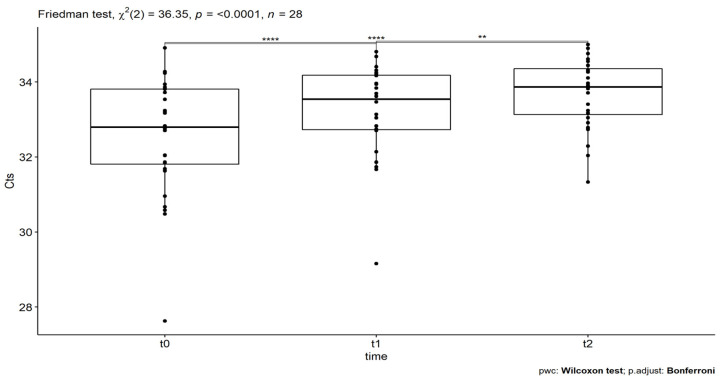
The boxplots of Ct measurements for Lyme disease at the different time points are t0 (before the first SOT administration), t1 (after the first treatment administration), and t2 (after the second treatment administration). The asterisks denote significance codes, namely ‘****’ stands for *p*-value < 0.0001 and ‘**’ stands for *p*-value < 0.01, after Bonferroni correction. The increases in the values of Ct were found to be statistically significant.

The statistically significant differences are related to increases in the Cts’ values. More analytically, Figure 2 shows the boxplots of Lyme disease measurements (Cts), where the time points of the SOT administrations are on the x-axis and the Cts on the y-axis. The asterisks denote significance codes, namely ‘****’ stands for *p*-value < 0.0001, ‘***’ stands for *p*-value < 0.001, ‘**’ stands for *p*-value < 0.01, and ‘*’ stands for *p*-value < 0.05, after Bonferroni correction. In particular, at t0, which corresponds to the initial Cts count before the first SOT administration, the mean value of Cts was found to be 32.6 (±0.29) for 95% CI. At t1 and t2, which correspond to the Cts counts after the first and second SOT administration, the mean values of Cts were found equal to 33.2 (±0.23) and 33.7 (±0.18), respectively, for 95%CIs. As shown, the increases in the values of Cts were found to be statistically significant in all cases. 

### 3.2. Herpes Viruses (HSV) 

In this case, Cts were measured four times from 28 patients with a median age of 53 years. The application of the Shapiro–Wilk test, in this case, revealed that the normality was not violated and, hence, the application of the one-way repeated ANOVA measures was feasible. In addition, the hypothesis of sphericity was tested using Mauchly’s test, revealing that the assumption of sphericity has been violated (*p* = 0.016 < 0.05), as shown in Table 3. Therefore, the Geisser–Greenhouse sphericity correction was applied. In this case, the epsilon (ε) was found equal to 0.78 < 1. After the sphericity corrections, the mean of Cts was found to be significantly statistically different, as depicted in Table 3 (*p* = 1.11 × 10^−7^ < 0.05) at different time points. The estimated F-statistic value was 18.54, while a small-size effect was found since the generalized effect size (ges) is 0.157 [34]. For the determination of which pairs exhibit statistically significant differences, multiple comparison testing was applied. The p-values were adjusted using the Bonferroni multiple-test correction method and the results are shown in Table 3. In particular, statistically significant differences were found for time points t0–t2 pair’s comparison (*p* = 0.00095 < 0.05), t0-t3 pair’s comparison (*p* = 9.6 × 10^−6^ < 0.05), t1-t2 pair’s comparison (*p* = 0.045 < 0.05), t1-t3 pair’s comparison (*p* = 0.001 < 0.05) and t2-t3 pair’s comparison (*p* = 0.023 < 0.05), but not for t0–t1 pair’s comparison (*p* = 0.17 > 0.05).

**Figure 3:**
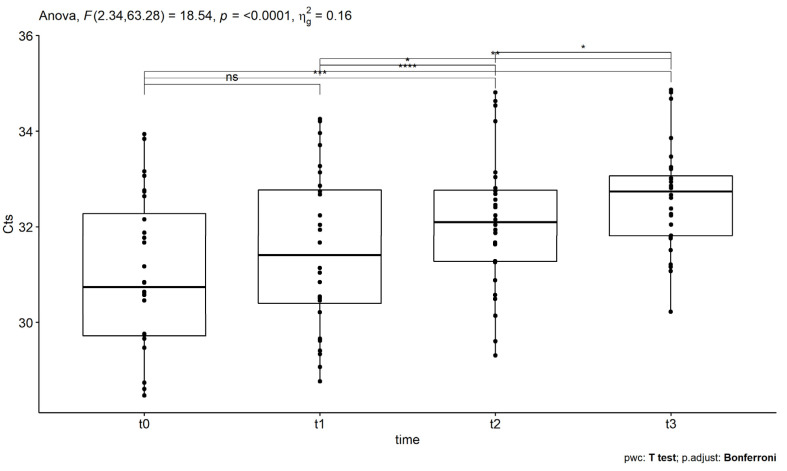
The boxplots of Ct measurements for the HSV virus at the different time points t0 (before the first SOT administration), t1 (after the first treatment administration), t2 (after the second treatment administration), and t3 (after the third treatment administration). The asterisks denote significance codes, namely ‘****’ stands for *p*-value < 0.0001, ‘***’ stands for *p*-value < 0.001, ‘**’ stands for *p*-value < 0.01, ‘*’ stands for *p*-value < 0.05, and ‘ns’ stands for non-significant, after Bonferroni correction. Increases, after the second and third SOT administrations, were found to be statistically significant.

The statistically significant differences are also related to increases in the Cts’ values. Specifically, Figure 3 shows the boxplots of HSV disease measurements (Cts), where the time points of the corresponding SOT administrations are on the x-axis and Cts on the y-axis. The asterisks denote significance codes, after Bonferroni corrections. At t0, which corresponds to the initial measurement before the first SOT administration, the HSV Cts have a mean value of 31.0 (±0.31) for 95%CI. At t1, which corresponds to HSV Cts after the first SOT treatment, the mean value was found equal to 31.5 (±0.31) for 95%CI. At t2, which corresponds to the measure after the second administration, the Cts’ mean value was found to be 32.1 (±0.27) for 95%CI. Finally, at t3, the measure after the third administration, the mean value was found equal to 32.6 (±0.21) for 95%CI. As can be seen in Figure 3, large statistically significant increases in Ct values occur after the second and third administrations.

### 3.3. Epstein–Barr Virus (EBV) 

Concerning the EBV virus, four repeated measures of Cts were obtained from 59 patients with a median age of 57 years. In this case, the normality hypothesis has not been verified, so the Friedman rank test was applied. More specifically, the results, shown in Table 4, revealed statistically significant differences at the different time points with test statistic X2(3) = 129.41 and *p* = 7.25 × 10^−28^ (<0.05). The application of the pairwise Wilcoxon signed-rank test revealed that all pairs proved to be significantly different. In particular, as depicted in Table 4, statistically significant differences in EBV’s measures were found between t0 and t1 pair’s comparison (*p* = 5.92 × 10^−8^ < 0.05), t0 and t2 pair’s comparison (*p* = 4.43 × 10^−8^ < 0.05), t0 and t3 pair’s comparison (*p* =2.29 × 10^−9^ < 0.05), t1 and t2 pair’s comparison (*p* =3.01 × 10^−7^ < 0.05), t1 and t3 pair’s comparison (*p* =3.54 × 10^−9^ < 0.05), and t2 and t3 pair’s comparison (*p* =6.96 × 10^−9^ < 0.05). Finally, a large effect size is detected, with Kendal’s W = 0.73.

**Figure 4:**
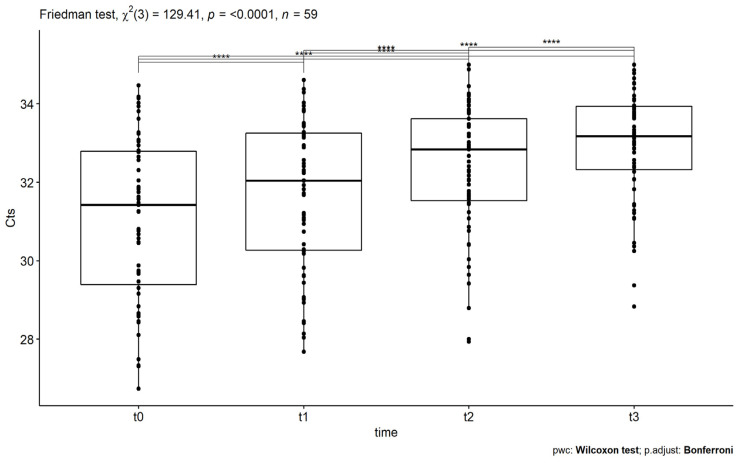
The boxplots of Ct measurements for the EBV virus at the different time points t0 (before the first SOT administration), t1 (after the first SOT treatment administration), t2 (after the second treatment administration), and t3 (after the third treatment administration). The asterisks denote significance codes, namely ‘****’ stands for *p*-value < 0.0001. All increases in the values are statistically significant.

The statistically significant differences are related to increases in the Cts’ values. More analytically, in Figure 4, the boxplots of EBV Cts are shown, where the time points of the SOT treatment administrations are on the x-axis, while the corresponding Cts are on the y-axis. At t0, which corresponds to the initial measurement before the first administration of SOT, the measurements were found to have an average value of 31 (±0.26) of 95% CI, while at time t1, which corresponds to measurements after the first administration, the mean value was found equal to 31.7 (±0.25) for 95% CI. Then, at t2, which corresponds to measurement after the second treatment, the mean value was found to be 32.4 (±0.21) for 95% CI, while in t3, which corresponds to measurement after the third administration, the mean value was found to be equal to 33 (±0.18) for 95% CI. As depicted in Figure 4, all increases are statistically significant.

## 4. Discussion

Epstein–Barr Virus (EBV) or Human Herpesvirus 4 (HHV4/HSV4) was discovered in 1964 when Epstein et al. studied cells cultured from Burkitt’s lymphoma [35]. Since then, EBV has been associated with various types of malignancies, such as nasopharyngeal carcinoma, non-Hodgkin’s lymphoma, and T-cell lymphomas [36]. The oncogenesis of EBV is linked to the deregulation of the cell cycle, through the interaction of EBV genes and host oncogenes, which lead, usually, to the promotion of G1/S phase transition and apoptosis inhibition [37]. Targeting the above latent genes is a therapeutic option for the inhibition of EBV proliferation. The latent membrane protein 1 (LMP1) can exhibit viral oncogenic properties, when unregulated, since it is known that it simulates the CD40 signaling pathway [38]. Antisense therapy against LPM1 or Epstein–Barr nuclear antigen 1 (EBNA1) can be beneficial for patients, either by inhibiting the proliferation of viruses or by affecting the EBV-transformed B-lymphocytes [39,40,41]. 

Human herpes simplex viruses 1 and 2 (HSV1/HSV2) are associated with a wide spectrum of clinical manifestations (encephalitis, corneal blindness, peripheral nervous system disorders) and constitute a major cause of morbidity and mortality worldwide [42]. HSV1 infects epithelial and neuronal cells [43] and HSV2 affects skin and mucous membranes, invading epithelial cells [44]. Previous literature and research data demonstrated that targeting specific genes of herpes simplex viruses might contribute to proliferation inhibition [45]. It is noteworthy that an antisense therapeutic option could be used also in strains resistant to antibiotics, used for HSV1 infections, such as acyclovir [46]. 

Lyme disease, also called Lyme borreliosis, is the most widely transmitted tick-borne infection. The bacterial spirochete Borrelia burgdorferi is the major cause of Lyme disease and is transmitted by the bite of an Ixodes genus stick. Other Borrelia species that can cause Lyme disease are Borrelia afzelii and Borrelia garinii, while many others have been identified in specific areas [47]. Upon infection, there are three different disease stages: early localized, early disseminated, and late stage. During the early stage, patients might present erythema migrans and a low-grade fever, while in stage two, patients may exhibit general malaise, fever, neurological features, muscle pain, and cardiac symptoms. Approximately one-fifth of patients present central nervous system (CNS) involvement, including encephalopathy, meningitis, and cranial nerve neuropathy. The last stage, which may occur for months or years, includes neurological and rheumatological features [48]. The treatment management of Lyme disease patients is not always easy and depends on many parameters, including patient age, stage, and features presented post-infection. Antisense oligonucleotides could also be used in Lyme disease. Knockdown of particular proteins, such as selenoprotein K, leads to the depletion of Borrelia burgdorferi within the tick host Ixodes scapularis, indicating that there is a wide range of therapeutics for siRNA molecules [49]. 

RNA interference as a therapeutic option is not a novel one. In 1998, the FDA licensed Fomivirsen (brand name Vitravene), which has been developed by Isis Pharmaceutical and is an antisense oligonucleotide for the treatment of cytomegalovirus retinitis (CMV) [50]. Other antisense-based drugs are approved, mainly for the treatment of neurological diseases, including Duchenne muscular dystrophy (DMD), spinal muscular atrophy (SMA), and familial amyloid polyneuropathy [51]. The above drugs consisted of modified oligonucleotides, since unmodified ones are highly unstable due to nuclease digestion. The modifications vary and might be the replacement of the non-linking with sulfur in the phosphodiester linkages or modifications on the 2′ position of the ribose ring [52,53]. Through intravenous administration, the bioavailability is maximized, which, in turn, means rapid distribution in specific organs, while coupling with other chemical groups provides the ability to bind to other organs and tissue as well [54,55]. 

The present study aims to provide a first, preliminary evaluation of the use of siRNAs in patients suffering from Lyme disease as well as from Epstein–Barr and Herpes simplex viruses HSV1/2. The results based on inferential statistics, particularly on one-way repeated measures ANOVA and its non-parametric version, namely the Friedman test, revealed that SOT therapy is related to statistically significant increases in Cts, after repeated SOT administrations. In particular, the analysis of data from 28 patients with Lyme disease, based on Friedman’s test, showed statistically significant increases (adjusted *p*-value < 0.05) after one and two SOT administrations, which are related to a large effect size since Kendall’s W was found equal to 0.65. In addition, the analysis of data from 28 patients with HSV disease, based on one-way repeated measures ANOVA test, showed statistically significant increases (adjusted *p*-value < 0.05) after two and three SOT administrations, but not after the first one. For this dataset, a small-size effect was detected, since the generalized effect size (ges) was found to be equal to 0.157. Finally, the analysis of data from 59 patients with EBV disease, based on Friedman’s test, showed statistically significant increases (adjusted *p*-value < 0.05) after one, two, and three SOT administrations. These statistically significant increases are connected with a large effect size, since Kendal’s W was found equal to 0.73.

## 5. Conclusions

Taking everything into consideration in terms of the preliminary data, SOT might be beneficial for patients with Lyme disease or infections by viruses, such as EBV and HSV. SOT is safe and specific for every target and can be used in many cases where a few drugs cannot be used. More samples need to be tested to implement SOT in the clinical routine; however, the preliminary inferential statistical data are encouraging for these infections.

## Figures and Tables

**Table 1:** Primer sequences for the different microorganisms.

Microorganism	Primer	Sequence (5′-3′)	Reference
Epstein–Barr Virus	EBNA-1 F	GAGCGGGGAGATAATGTACA	[14]
	EBNA-1 R	TAAAAGATGGCCGGACAAGG	
	EBNA2-F	AGGGATGCCTGGACACAAGA	
	EBNA2-R	TTGTGACAGAGGTGACAAAA	
	VCA F	CGGTGTAACTACCCGCAATG	[15]
	VCA R	CGTGGTCGTGTTCCCTCA	
	EA F	AGGACCTACGCTGCCCTAGA	[16]
	EAR	AAAACATGCGGACCACCAGC	
Herpes simplex virus 1	gG1 F	TTGGTTCTTGTCGGTGTATCG	In house
	gG1 R	GGCGTGGTAAGGCTGATG	
Herpes simplex virus 2	gG2 F	GACCCAAAGACGCACCCA	
	gG2 R	CGCCCTCAAACTCCTCGG	
Borrelia burgdorferi	OspA F	GTTTTGTAATTTCAACTGCTGACC	[17]
	OspA R	GCCATTTGAGTCGTATTGTTGTACTG	
	OspB F	AACAAGATCAAACGGAACTACACT	In house
	OspB R	CCGACTACAAGACTTCCTTCAAG	

**Table 2:** The statistical results of the non-parametric tests (Friedman, Wilcoxon) for Lyme disease. The asterisks denote significance codes, namely ‘****’ stands for *p*-value < 0.0001 and ‘**’ stands for *p*-value < 0.01, after Bonferroni correction.

Friedman Rank Test						
Response	n	Friedman Chi-Squared Statistic		df	*p*	
Ct	28	36.35		2	1.28 × 10−8	
Wilcoxon Pairwise Comparisons						
Response	statistic	*p*	*p*	*p*.signif	pairs	
Ct	5	1.04 × 10−5	3.12 × 10−5	****	t0	t1
	19	2.29 × 10−6	6.87 × 10−6	****	t0	t2
	39	0.0005	0.002	**	t1	t2
Friedman Test Effect Size						
Response	n		Kendall’s W		df	
Ct	28		0.65		2	

**Table 3:** The statistical results of the parametric tests (ANOVA, T-test) for Herpes Viruses. The asterisks denote significance codes, namely ‘****’ stands for *p*-value < 0.0001, ‘***’ stands for *p*-value < 0.001, ‘**’ stands for *p*-value < 0.01, ‘*’ stands for *p*-value < 0.05, and ‘ns’ stands for non-significant, after Bonferroni correction.

Mauchly’s Test for Sphericity							
Effect		Mauchly’s Statistic	*p*			*p* < 0.05	
time		0.58	0.016			*	
One way repeated measures ANOVA							
Effect	DFn	DFd	F	*p*	*p* < 0.05	ges	
time	2.34	63.28	18.54	1.11 × 10^−7^	****	0.157	
*t*-test pairwise comparisons							
Response	t-statistic	df	*p*	*p*	*p*.signif	pairs	
Ct	−2.31	27	0.028	0.17	ns	t0	t1
	−4.38	27	0.0001	0.000948	***	t0	t2
	−6.10	27	1.6 × 10^−6^	9.6 × 10^−6^	****	t0	t3
	−2.89	27	0.007	0.045	*	t1	t2
	−4.22	27	0.0002	0.001	**	t1	t3
	−3.16	27	0.004	0.023	*	t2	t3

**Table 4:** The statistical results of the non-parametric test (Friedman, Wilcoxon) for EBV. The asterisks denote significance codes, namely ‘****’ stands for *p*-value < 0.0001.

Friedman Rank Test						
Response	n	Friedman X2 Statistic		df	*p*	
Ct	59	129.41		3	7.25 × 10^−28^	
Wilcoxon pairwise comparisons						
Response	statistic	*p*	*p*	*p*.signif	pairs	
Ct	125	9.87 × 10^−9^	5.92 × 10^−8^	****	t0	t1
	118.5	7.39 × 10^−9^	4.43 × 10^−8^	****	t0	t2
	55	3.82 × 10^−10^	2.29 × 10^−9^	****	t0	t3
	151	5.01 × 10^−8^	3.01 × 10^−7^	****	t1	t2
	64	5.9 × 10^−10^	3.54 × 10^−9^	****	t1	t3
	60	1.16 × 10^−9^	6.96 × 10^−9^	****	t2	t3
Friedman Test Effect Size						
Response	n		Kendall’s W			df
Ct	59		0.73			3

## Data Availability

The datasets used and/or analyzed during the current study are available from the corresponding author upon reasonable request.
